# Intratracheal Budesonide Combined With Surfactant for Pulmonary Hemorrhage in a Preterm Neonate: A Case Report

**DOI:** 10.7759/cureus.96306

**Published:** 2025-11-07

**Authors:** Saeka Matsubara, Aiko Tanimoto, Masahiko Sato

**Affiliations:** 1 Department of Neonatology, Tokyo Women's Medical University, Yachiyo Medical Center, Yachiyo, JPN; 2 Department of Pediatrics, Juntendo University, Urayasu Hospital, Urayasu, JPN

**Keywords:** acute respiratory distress syndrome, budesonide, extremely premature infants, pulmonary hemorrhage, surfactant

## Abstract

Intratracheal budesonide combined with surfactant has been recently reported to be effective in the treatment of respiratory distress syndrome (RDS), acute respiratory distress syndrome (ARDS), and other conditions associated with reduced surfactant activity and lung inflammation. This report presents the first case of intratracheal budesonide using surfactant for acute pulmonary hemorrhage and severe respiratory failure in an extremely low birthweight infant. A 251 g preterm infant born at the 24th gestational week was intubated, received one dose of intratracheal surfactant for the treatment of RDS on her first day of life. The patient’s respiratory condition was stable using assist/control mode ventilation with a fraction of inspired oxygen (FiO_2_) of 0.28, and the oxygenation index (OI) was between 1 and 2 on her first day of life. However, she developed severe pulmonary hemorrhage at 30 hours after birth. Because pulmonary hemorrhage persisted and the maximum oxygenation index was 42.5 at 45 hours after birth, despite high-frequency oscillatory ventilation (HFOV) with high mean airway pressure and high concentration of inhaled oxygen (FiO_2_=0.9), we administered intratracheal budesonide (0.25 mg/kg/dose) combined with surfactant (80 mg/kg/dose) at 54 hours of age. As a result, her respiratory conditions successfully improved, and the OI reduced stepwise to 19.5 at 56 hours of age and 5.3 at 66 hours of age. Respiratory acidosis also improved rapidly, and FiO_2_ could be reduced to 0.4 within 24 hours of treatment. No pulmonary hemorrhage was observed after 62 hours of age. She no longer required any respiratory support or oxygen therapy at 136 days of age. This case suggests that intratracheal budesonide with surfactant can be a novel therapeutic option for acute respiratory failure due to pulmonary hemorrhage.

## Introduction

Pulmonary hemorrhage in premature infants is associated with a poor prognosis and a high mortality rate. Previously reported therapeutic options for pulmonary hemorrhage include intratracheal surfactant, ventilatory support (high-frequency oscillatory ventilation (HFOV) and high positive end-expiratory pressure), intratracheal epinephrine, intravenous tolazoline, and management of coagulopathy [1].

The deterioration of respiratory status following pulmonary hemorrhage is significantly caused by the leakage of blood components into the alveolar space and the inactivation of surfactant [2]. Intratracheal surfactant administration for pulmonary hemorrhage has been reported to rapidly improve oxygenation and ventilation [3-5]. Additionally, intratracheal budesonide with surfactant has recently been noted to be beneficial in conditions associated with surfactant inactivation and lung inflammation, such as respiratory distress syndrome (RDS) [6,7] and acute respiratory distress syndrome (ARDS) [8].

Budesonide is pathologically expected to provide anti-inflammatory effects and prevent surfactant inactivation by reducing the release of proinflammatory cytokine/chemokines from lung macrophages activated by secretory phospholipase A2, which inhibits surfactant activity [9]. In clinical practice, the combination of budesonide and surfactant for RDS has been reported to improve oxygenation and pulmonary ventilation immediately after administration compared to surfactant alone [7], and to be effective even for refractory surfactant deficiency secondary to sepsis [8]. However, there are no reports regarding secondary RDS due to pulmonary hemorrhage. This report presents the first case of an extremely low birth weight infant with severe respiratory failure due to acute pulmonary hemorrhage who was successfully treated with intratracheal administration of budesonide plus surfactant.

## Case presentation

A 251 g female infant was delivered via emergency cesarean section at 24th gestational week to a 37-year-old mother, who had been hospitalized for severe hypertensive disorders of pregnancy and intrauterine growth restriction and had received two doses of betamethasone at the 23rd gestational week. The Apgar scores at one and five minutes were 3 and 7, respectively.

**Figure 1:**
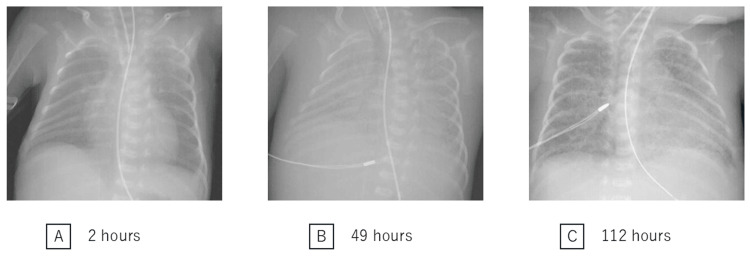
Chest radiograms (A) Two hours after birth (immediately after the first surfactant administration for RDS treatment): The response to the first surfactant was favorable, and the lung expansion was achieved. (B) At 49 hours after birth (five hours before the budesonide-surfactant administration): The chest radiogram findings showed a white-out appearance, suggesting massive pulmonary hemorrhage. (C) At 112 hours after birth (58 hours after the budesonide-surfactant administration): The chest radiogram findings showed improvement in lung aeration. RDS: respiratory distress syndrome

**Figure 2:**
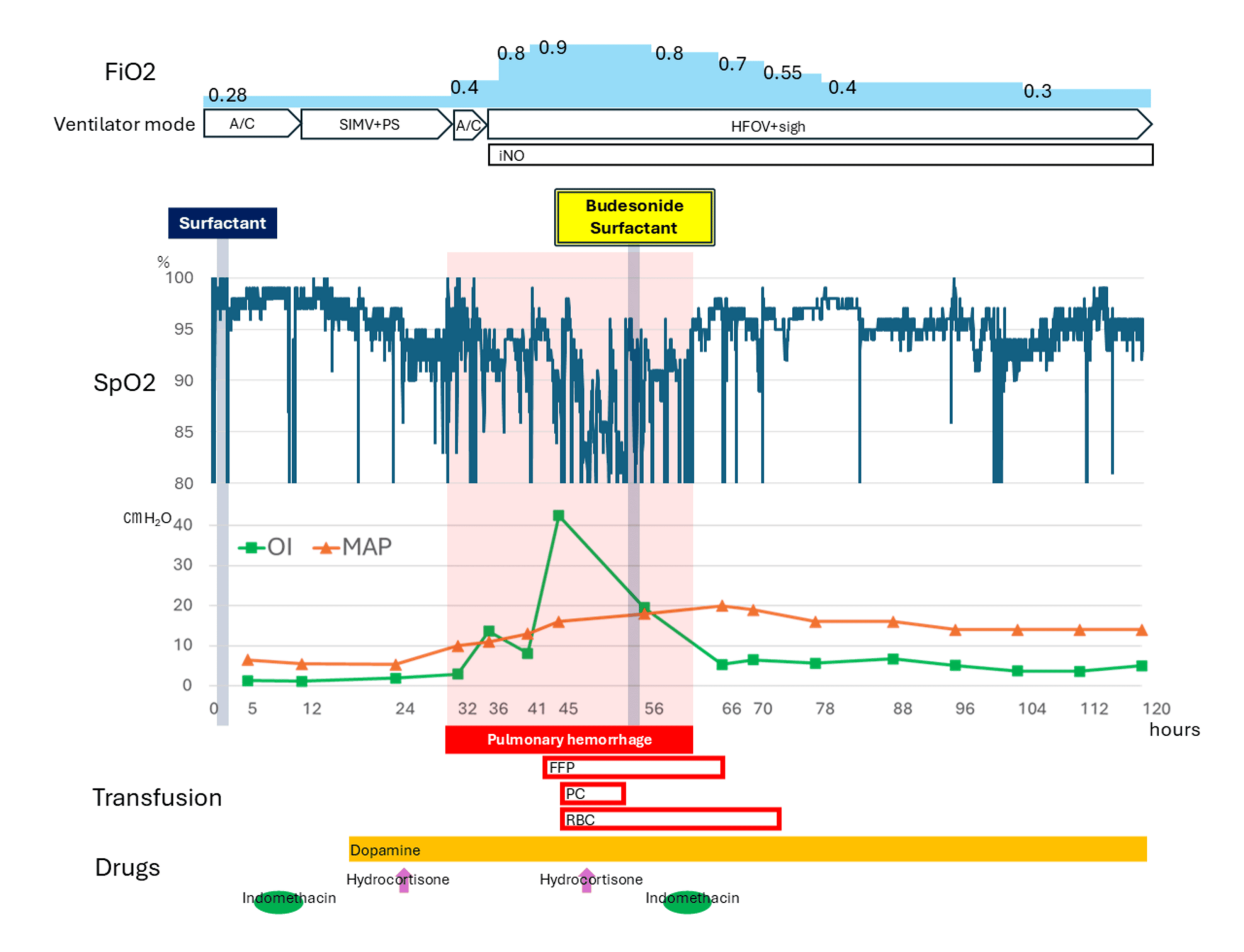
Changes in oxygenation index (OI), measured MAP (mean airway pressure), percutaneous arterial oxygen saturation (SpO2), and fraction of inspired oxygen (FiO2) ※OI (cmH_2_O/mmHg) = measured MAP×FiO_2_×100/PaO_2_; ※ SpO_2_ values were extracted from the biometric monitor's records at one minute intervals; ※ Time is indicated as hours after birth. A/C: assist/control; SIMV: synchronized intermittent mandatory ventilation; PS: pressure support; HFOV: high frequency oscillatory ventilation; iNO: inhaled nitric oxide; FFP: fresh frozen plasma; PC: platelet concentrate; RBC: red blood cells

The infant was intubated, received one dose of surfactant for the treatment of RDS, and was ventilated using assist/control mode (Babylog VN500, Draeger, Inc., Tokyo, Japan). The patient responded well to surfactant therapy, and the lungs expanded adequately (Figure 1A). Indomethacin at 0.1 mg/kg was administered eight hours after birth to manage the patent ductus arteriosus (PDA). Intravenous dopamine was initiated at 17 hours of age, and hydrocortisone at 1 mg/kg was injected at 24 hours of age due to hypotension. The patient’s respiratory condition was stable using assist/control mode ventilation with a fraction of inspired oxygen (FiO2) of 0.28; the oxygenation index (OI) was between 1 and 2 on her first day of life (Figure 2).

However, pulmonary hemorrhage was observed at 30 hours of age, while echocardiograms indicated severe tricuspid regurgitation and pulmonary hypertension. She developed acute worsening of oxygenation with a FiO_2_ of 0.8 at 36 hours of age (OI: 13.5, mean airway pressure (MAP): 11) (Figure 2), and the blood gas analysis revealed respiratory acidosis at 32 hours of age (Table 1).

**Table 1:** Complete blood count and blood gas analysis Time is indicated as hours after birth.

Parameter	24 hours	32 hours	45 hours	56 hours	66 hours	78 hours	Reference range	Unit
White blood cell count	4.63	4.28	4.08	2.7	2.76	2.11	5.0-21.0	10^9^/L
Hemoglobin	115	94	74	54	96	122	142-172	g/L
Red blood cell count	2.74	2.15	1.79	1.5	2.85	3.82	3.51-4.27	10^12^/L
Hematocrit	36.5	30.1	23.9	18.3	28.7	35.8	40-50	%
Platelet count	147	101	74	86	84	53	133-229	10^9^/L
Prothrombin time-international normalized ratio	/	1.75	1.72	/	1.35	1.36	0.7-2.1	
Activated prothrombin time	/	>150.0	139.2	/	57.5	61.2	24.5-68.4	seconds
Fibrinogen	/	61	60	/	118	109	150-373	mg/dL
pH	7.329	7.099	7.07	7.246	7.244	7.271	7.35-7.45	
Arterial oxygen partial pressure	67.7	140	33.9	73.8	266	128	83-108	mmHg
Arterial carbon dioxide partial pressure	37.1	73.8	73.6	40.4	40.4	41.1	40-50	mmHg
Na^+^(Sodium) concentration	147	152	151	151	153	157	136-146	mmol/L
K^+^(Potassium) concentration	5.3	5.8	6.2	5.3	5.1	4.3	3.5-5.0	mmol/L
Ca^2+^(Calcium) concentration	1.65	1.62	1.41	1.20	1.17	1.36	1.15-1.29	mmol/L
Cl^-^(Chloride) concentration	118	122	123	124	124	127	98-106	mmol/L
Base excess	-5.9	-8.0	-8.8	-9.0	-9.5	-7.7	-3.0-3.0	mmol/L
Standard base excess	-5.8	-6.6	-8.4	-9.0	-9.1	-7.3	-3.0-3.0	mmol/L
HCO_3_^-^(Bicarbonate) concentration	19	21.8	20.4	16.9	16.8	18.3	22-27	mmol/L
Anion gap	9.4	8.3	7.8	10.2	11.7	11.7	12-16	mmol/L
Lactic acid	40	20	26	49	56	46	4.5-14.4	mg/dL

The ventilator settings were immediately changed to HFOV with inhalation of nitric oxide. Transfusion of plasma derivatives, platelets, and red blood cells was required in sequence because of severe anemia, platelet deficiency, and blood coagulation factor deficiency (Figure 2, Table 1). The setting MAP was gradually increased, and the “Sigh” option was added to HFOV, resulting in a measured MAP of 16 cmH_2_O at 43 hours of age. Despite these measures, her respiratory status did not improve, and the OI reached 42.5 at 45 hours of age (Figure 2). Intravenous hydrocortisone at 4 mg/kg was also administered at 46 hours of age. The ventilator settings were further increased to ensure adequate ventilation, with the maximum measured MAP reaching 19cmH_2_O at 51 hours of age (setting MAP: 13 cmH_2_O, sigh peak inspiratory pressure: 38 cmH_2_O, sigh inspiration time: 0.65 seconds, sigh rate: 20 times per minute). The chest X-ray showed a white-out appearance, suggesting massive pulmonary hemorrhage (Figure 1B).

As pulmonary hemorrhage persisted and severe desaturation occurred frequently despite the high concentration of inhaled oxygen (FiO_2_=0.9), we determined to administer intratracheal surfactant plus budesonide at 54 hours of age with parental consent. Surfactant at 80 mg (Surfacten®; Mitsubishi Tanabe Pharma, Osaka, Japan) was mixed with 0.25 mg/kg budesonide (Pulmicort Respules®; AstraZeneca plc, Cambridge, United Kingdom) and administered intratracheally using the same technique as surfactant alone. As a result, her respiratory conditions successfully improved, and the OI reduced stepwise to 19.5 at 56 hours of age (two hours after the budesonide therapy), 5.3 at 66 hours of age, and 3.7 at 104 hours of age, respectively (Figure 2). Chest X-ray findings also showed improvement in lung aeration (Figure 1C). Respiratory acidosis also improved rapidly, and FiO_2_ could be reduced to 0.4 within 24 hours of treatment. No pulmonary hemorrhage was observed after 62 hours of age.

A second dose of indomethacin (0.24 mg/kg) was given two hours after budesonide surfactant therapy for the treatment of PDA, which functionally closed on the third day of life. Her respiratory status continued to stabilize with HFOV, and she was switched to neurally adjusted ventilatory assist (NAVA) at 49 days of age and extubated at 86 days of age. She was managed with noninvasive ventilation (NIV)-NAVA until day 91, and was supported by high-flow nasal cannula therapy and use of oxygen until day 136 of life. She was diagnosed with chronic lung disease grade 3 (NIH 2018 definition), but was discharged without respiratory support or oxygen therapy at 194 days of age. The patient required dopamine until a month of age and hydrocortisone from 10 to 56 days of age for the late-onset circulatory collapse. Otherwise, she was diagnosed with severe retinopathy of prematurity (ROP), requiring bilateral intravitreal injections of ranibizumab. She did not develop intraventricular hemorrhage or necrotizing enterocolitis (NEC).

## Discussion

We reported the first successful treatment using intratracheal budesonide and surfactant for severe respiratory failure due to pulmonary hemorrhage in a very preterm infant. In recent years, conditions in neonates arising from secondary impairment of surfactant due to sepsis, pneumonia, aspiration, or pulmonary hemorrhage are now defined as neonatal acute respiratory distress syndrome (NARDS), as in adults and children. NARDS is distinguished from RDS, which is characterized by primary surfactant deficiency [10]. Epidemiologic studies reveal that pulmonary hemorrhage accounts for 10.5% of NARDS [11].

Pulmonary hemorrhage leads to the leakage of blood components into alveolar spaces, causing surfactant inactivation through interactions with plasma proteins such as albumin and fibrinogen and cell-free hemoglobin [2]. This process contributes to the development of NARDS. In NARDS, the upregulation of secretory phospholipase A2 (sPLA2) produced by lung macrophages also plays an important role in surfactant inactivation [12,13]. sPLA2 hydrolyzes glycerophospholipids, the major component of biological membranes, generating lysophospholipids and free fatty acids, both of which inactivate surfactant [13]. Free fatty acids also act as substrates for arachidonic acid, triggering the arachidonic acid cascade and promoting further inflammation [13]. Lysophosphatidylcholine (LPC), a lysophospholipid, can disrupt the packing of the phospholipid film at the air-liquid interface and inhibit reduction of surface tension during dynamic compression, while plasma proteins such as albumin and hemoglobin inhibit surfactant function by a competition between the proteins and phospholipids for space at the air-liquid interface [2]. Thus, the plasma protein-induced surfactant inactivation is abolished at sufficiently high surfactant phospholipid concentrations [14]; however, sPLA2-induced lysophospholipid inhibition of surfactant function is independent of the phospholipid concentration [15].

Interestingly, Amizuka et al. collected lung effluent from neonates with pulmonary hemorrhage and found that 81% (22/27) of these infants actually had inhibition activity against surfactant [4]. They also reported that surfactant inhibition was independently associated with early onset of pulmonary hemorrhage and lower surfactant protein-A (SP-A)/Alb ratio. SP-A is a hydrophilic surfactant protein that not only supports both adsorption and diffusion of surfactant at the air-liquid interface, but also has anti-infective and anti-inflammatory properties by down-regulating and inactivating sPLA2 [12]. It has been demonstrated in vitro that SP-A can counteract the surfactant inhibitory effect of plasma proteins [16], while the effect of SP-A itself is insufficient against LPC [15].

Budesonide is noted for its surfactant-activating and anti-inflammatory effects. Budesonide inhibits the release of tumor necrosis factor-alpha (TNF-α), interleukin (IL)-6, and IL-8 by blocking gene expression in sPLA2-stimulated lung macrophages and therefore may provide a therapeutic strategy for diseases associated with enhanced release of sPLA2 [9]. Budesonide is also reported to inhibit transforming growth factor beta (TGF-β) signaling and to increase expression of SP-A and surfactant protein-B (SP-B), which has properties to reduce surfactant hydrolysis caused by sPLA2 [17]. Therefore, intratracheal administration of budesonide with surfactant for pulmonary hemorrhage is expected to prevent surfactant inactivation, directly by supplementing the amount of surfactant and indirectly by increasing SP-A expression and decreasing sPLA2 expression.

Pulmonary hemorrhage is a condition where plasma proteins leak into the alveolar space, readily inactivating surfactant. Since plasma protein inhibition of surfactant is competitive, exogenous pulmonary surfactant administration is considered effective. However, when pulmonary hemorrhage progresses to NARDS, treatment with exogenous　surfactant alone is likely to fail. This is due to the combined effects of macrophage stimulation and cytokine release mediated by sPLA2, the inflammatory cascade triggered by simultaneously generated free fatty acids, and increased production of LPC, which directly inhibits pulmonary surfactant. In such severe conditions, it is considered reasonable to combine surfactant administration with budesonide, which suppresses sPLA2 responsible for supplying LPC. Additionally, surfactant may serve as an efficient drug delivery agent to deliver budesonide in a more homogenous and peripheral lung distribution [18].

To date, no randomized controlled trials (RCTs) have been conducted to evaluate the efficacy of surfactant administration specifically for neonatal pulmonary hemorrhage [19], while retrospective observational studies have reported improved OI with surfactant administration [3,4,5,20]. Yen et al. reported that patients administered surfactant for pulmonary hemorrhage showed significantly earlier improvement in oxygenation compared to patients not administered surfactant [5]. Amizuka et al. demonstrated that the pulmonary fluid of patients unresponsive to surfactant therapy contained low levels of disaturated phosphatidylcholine (DSPC) prior to treatment [4]. Considering that sPLA2 hydrolyzes the glycerophospholipid DSPC to produce LPC [21], this may have reflected LPC's inhibition of exogenous surfactant monotherapy for pulmonary hemorrhage. Furthermore, in an RCT by Rong et al. that administered surfactant alone for pneumonia-induced NARDS, the surfactant group showed significantly lower OI at 4 and 12 hours compared to the non-treatment group, but the difference was no longer significant at 24 hours [22]. This suggests that the effect of surfactant may be limited in conditions where sPLA2 is activated.

Budesonide combined with intratracheal surfactant is currently reported to be effective in cases of RDS [6,7] and ARDS [8]. Yeh et al. conducted an RCT involving 265 very low birth weight infants with RDS and found that the budesonide-surfactant group had significantly fewer surfactant doses and significantly lower interleukin levels (IL-1, IL-6, IL-8) in tracheal aspirate compared to the surfactant-only group [7].

Furthermore, several studies have reported that budesonide-surfactant therapy for RDS not only improves short-term respiratory status but also reduces the incidence of bronchopulmonary dysplasia (BPD) [6,7]. Yeh et al. reported intratracheal administration of surfactant/budesonide significantly decreased the incidence of BPD or death compared to surfactant alone [7]. In a meta-analysis on RDS, Yi et al. suggested that budesonide-surfactant therapy significantly reduced duration of ventilation, hospitalization, and incidence of BPD compared to surfactant alone [6]. Although the PLUSS (Preventing Lung Disease Using Surfactant + Steroid) double-blind randomized clinical trial conducted in 21 neonatal units in four countries suggested that early intratracheal budesonide mixed with surfactant had little to no effect on survival free of BPD [23]. The effects of budesonide surfactant therapy on the incidence of BPD are still unclear, and further research is needed to determine the role of this treatment. The Budesonide in Babies trial (NCT04545866), enrolling at centers in the United States, which includes only extremely preterm infants, is ongoing [24].

Up to now, no RCTs have been conducted on the efficacy of budesonide combined with surfactant therapy for NARDS. Previous studies on budesonide-surfactant used for RDS mentioned above did not report a subgroup that developed pulmonary hemorrhage [6,7]. Deliloglu et al. reported that intratracheal administration of budesonide-surfactant improved respiratory status in two very low birth weight infants who were difficult to treat with surfactant alone for ARDS due to sepsis [8]. Gunes et al. reported improved oxygenation with budesonide combination therapy in a retrospective case series of NARDS secondary to late-onset sepsis [25].

In this case, when pulmonary hemorrhage occurred, we attempted management with a high MAP strategy on the ventilator, but both oxygenation and respiratory acidosis worsened. Although anticoagulant therapy was administered concomitantly, pulmonary hemorrhage requiring transfusion persisted. Considering not only the leakage of plasma proteins and red blood cells into the alveolar space but also the progression of the inflammatory cascade and increased LPC production by sPLA2, intravenous hydrocortisone was also attempted, but the response was limited. We judged that treatment with surfactant alone would be difficult and opted for combination therapy with budesonide, referencing the report by Deliloglu et al. [8]. Consequently, the patient's respiratory status stabilized promptly after the administration, and the treatment was successful.

The first limitation of this report is whether budesonide surfactant therapy was effective for pulmonary hemorrhage and respiratory failure. Despite the limited efficacy of high-pressure ventilator settings and intravenous steroid administration in this case, rapid improvement in respiratory status was observed after budesonide combination therapy, and this favorable condition was maintained. Considering that previous studies reported effects within one to six hours after administration [3,4,5,20], and that the time to onset of effect in this case was consistent with this, we concluded that budesonide-surfactant administration was effective in this patient. Following the sudden worsening of oxygenation, we explored treatment options while further increasing ventilator settings, which resulted in a time lag before deciding to administer budesonide-surfactant. Although it is possible that partial alveolar recruitment achieved through ventilator setting adjustments during this period may have already caused the OI to decline before budesonide-surfactant administration, we assess that budesonide-surfactant contributed to the subsequent stabilization and improvement. The second limitation is that it is not strictly clear whether the combination with budesonide was necessary. However, in life-threatening critical situations, there is no time to attempt surfactant monotherapy. The third limitation is that this report concerns a single case of an extremely low birth weight infant of a size rarely encountered, limiting its generalizability. Future studies involving larger case series and randomized trials are necessary to determine the efficacy of budesonide-combined surfactant therapy for pulmonary hemorrhage.

The lack of adequate studies on the short and long-term prognosis of intratracheal administration of budesonide should be of concern. In the meta-analysis by Yi et al., there were no significant differences in the incidence of ROP, NEC, PDA, or sepsis between the two groups [6]. Yeh et al. also reported no significant differences in the incidence of neuromotor dysfunction, mental development index, psychomotor development index, weight, or height between the groups at follow-up [7]. In addition, Roberts et al. reported that intratracheal budesonide administered with surfactant was not detected in brain tissues in preterm lambs [26].

## Conclusions

We have successfully treated a very low birth weight infant with severe respiratory failure due to pulmonary hemorrhage using intratracheal budesonide administration with surfactant. In severe pulmonary hemorrhage with advanced lung injury, surfactant therapy alone may be unresponsive or provide only temporary effects. This case suggests that adjuvant therapy combining budesonide with surfactant treatment could be one therapeutic option for acute respiratory failure due to pulmonary hemorrhage. However, this report is based on a rare single case, and this is not a formal recommendation. Further case series and prospective studies are warranted to determine the efficacy of intratracheal budesonide with surfactant in the treatment of severe pulmonary hemorrhage.
